# Embryonic Heart Morphogenesis from Confocal Microscopy Imaging and Automatic Segmentation

**DOI:** 10.1155/2013/293069

**Published:** 2013-12-23

**Authors:** Hongda Mao, Megan Gribble, Arkady M. Pertsov, Pengcheng Shi

**Affiliations:** ^1^Computational Biomedicine Laboratory, Rochester Institute of Technology, Rochester, NY 14623, USA; ^2^Department of Pharmacology, SUNY Upstate Medical University, Syracuse, NY 13210, USA

## Abstract

Embryonic heart morphogenesis (EHM) is a complex and dynamic process where the heart transforms from a single tube into a four-chambered pump. This process is of great biological and clinical interest but is still poorly understood for two main reasons. On the one hand, the existing imaging modalities for investigating EHM suffered from either limited penetration depth or limited spatial resolution. On the other hand, current works typically adopted manual segmentation, which was tedious, subjective, and time consuming considering the complexity of developing heart geometry and the large size of images. In this paper, we propose to utilize confocal microscopy imaging with tissue optical immersion clearing technique to image the heart at different stages of development for EHM study. The imaging method is able to produce high spatial resolution images and achieve large penetration depth at the same time. Furthermore, we propose a novel convex active contour model for automatic image segmentation. The model has the ability to deal with intensity fall-off in depth which is characterized by confocal microscopy images. We acquired the images of embryonic quail hearts from day 6 to day 14 of incubation for EHM study. The experimental results were promising and provided us with an insight view of early heart growth pattern and also paved the road for data-driven heart growth modeling.

## 1. Introduction

The heart is the first functioning organ in the embryo. Although the morphology of the heart changes dramatically during development where it transforms from a single tube into a four-chambered pump, the heart functions without interruption to serve the metabolic needs of the rapidly growing embryo [1]. Embryonic heart morphogenesis (EHM) is critically important for long-time survival, and any defects in the developmental mechanism during embryogenesis may result in congenital cardiac anomalies. In fact, congenital heart disease is relatively frequent which affects from 19 to 75 per 1000 births worldwide and has been an important cause of childhood morbidity and mortality [2]. Understanding EHM in normal and malformed hearts, therefore, has been of considerable clinical and biological interest.

Despite a large body of research in the last decades [3–8], EHM is still poorly understood mainly because of the complexity of the growing geometry and extremely small size of the developing heart. Thanks to the rapid development of imaging techniques, 3D reconstruction of embryonic hearts from biomedical images has dramatically improved our ability to visualize EHM. Several imaging modalities have been proposed for the study of EHM; however, each of them has its own limitations. Histological sectioning was one of the most widely used approaches for rendering 3D structure of the developing heart [6]. Nevertheless, it needed sophisticated manual alignment of all the sections which was difficult and labor-intensive and therefore left it only for lab researchers. Optical scanning techniques were also used for rendering 3D/4D volumes of embryonic hearts [7, 9], but low penetration depth limits their application in imaging late stages of embryonic heart development [8]. There were also other imaging modalities used for understanding EHM which, unfortunately, provided very limited spatial resolution [10]. Recently, micro-CT technique was used to image the chambers of embryonic hearts [8]. However, the sophisticated polymerization process ignored the structure of the peripheral luminal space which is actually very important for EHM understanding. Last but not least, all the aforementioned works adopted manual segmentation for 3D heart segmentation due to the lack of appropriate automatic segmentation approaches. Nevertheless, manual segmentation is tedious, subjective, and time consuming considering the complexity of the developing heart and high resolution images. Thus, an automatic image segmentation method is badly needed.

In view of the problems, we propose a new imaging approach for studying EHM, utilizing tissue optical immersion clearing and 3D confocal microscopy imaging, which can produce high spatial resolution images and achieve large penetration depth at the same time. Furthermore, considering the intensity fall-off in depth nature of confocal microscopy images, we propose a convex active contour model with image depth information for automatic image segmentation. A recently proposed Split Bregman method was used to minimize the objective function of the model [11, 12]. Embryonic quail hearts at different stages of development were scanned and segmented. Initial heart growth pattern was found through comparison of the structure of hearts at different stages of development. We also quantified the volume change of the whole heart and luminal space from day 6 to day 14 of incubation to provide an insight view of embryonic heart development. Furthermore, realistic continuous growth modeling of the living organs from data sparsely distributed in time has been an emerging field in biomedical field [13, 14], with potential applications to the analysis and prediction of evolving pathologic structures. Thus, this work can be also considered as the first step for data-driven heart growth modeling.

## 2. Methodology

The human heart becomes a four-chambered organ by approximately week 8, which is almost the same time the embryo can be visualized through ultrasound, a point that is too late to visualize EHM [15]. We chose quail embryos as our model to study EHM because of their rapid development and short embryonic gestation period (a hatch time of 16.5 days of incubation). Except for the time scale, the development of the quail heart parallels that of the human heart.

### 2.1. Image Acquisition

Optical imaging method has been widely used as a tool for clinical functional imaging owing to its unique informative features, simplicity, safety, and low cost compared to conventional X-ray, MRI, and ultrasound imaging. However, the main limitations of optical imaging techniques, including confocal microscopy, are low contrast and spatial resolution, as well as a small probing depth due to strong light scattering in tissue layers [16]. To utilize its strengths and overcome its weaknesses, we combined confocal microscopy imaging with tissue optical immersion clearing. Optical clearing technique has been used in many areas [16]. However, to the best of our knowledge, this is the first time to use optical clearing with confocal microscopy imaging for EHM study.

In this paper, all the experiments conformed to the *Guide for the Care and Use of Laboratory Animals* (NIH publication no. 85-23, revised 1996). Embryonic hearts were obtained after incubation of *Coturnix japonica* (GQF Manufacturing Co., Savannah, GA) or Japanese quail eggs to different stages of development. The hearts were stained with di-4-ANBDQBS which was voltage sensitive fluorescent dyes and then were dehydrated by a graded ethanol series. After dehydration, the hearts were cleared using a 1 : 2 benzyl alcohol to benzyl benzoate mixture. The cleared heart, which appeared virtually transparent, was stored in the clearing solution until imaging. For image acquisition, the cleared hearts were mounted in a special cuvette and scanned by a Zeiss LSM 510 confocal microscope, with its numerical aperture being equal to 0.5 and the radius of back-projected pinhole equal to 2.53 nm. The dye was excited at wavelength of 543 nm and fluorescence recorded the wave length above 560 nm using a long-pass filter. For more details of heart preparation and imaging, we refer the readers to [17].

**Figure 1:**
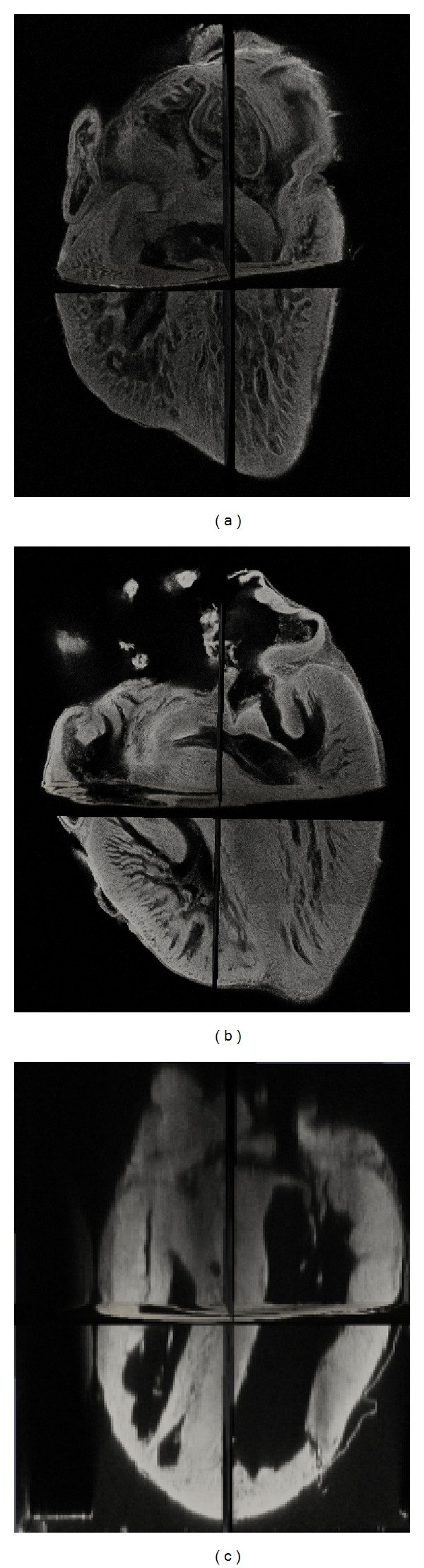
3D view of embryonic quail hearts. From left to right: day 6, day 8, and day 14.

High spatial resolution images were obtained after heart scanning, which had an intraslice pixel size of 1.75 *μ*m × 1.75 *μ*m and interslice pixel size 12.9000 *μ*m. In Figure 1, we present 3D view of three hearts at days 6, 8, and 14, respectively. From the images, the evolution of the luminal space of the heart from spongy structure to well-separated chamber structure can be clearly observed.

### 2.2. Image Formation

In a confocal microscope, a pinhole is used to reject most out-of-focus light. Thus, the amount of light reaching the detector is low, and the noise statistics can be well described by a Poisson process [18]. A general image formation model can be represented as the following equation:

(1) $$\begin{array}{c} I_{0}\left(x\right)=n\left(\left[h*I\right]\left(x\right)\right), \end{array}$$

where *x* ∈ *Ω* is a point in the image domain. *I*_0_ is an observed image. *I* is an ideal image. *h* is a point spread function (PSH). ∗ means convolution operation. *n* models the noise distribution. Based on our imaging setting, the PSF of the microscope is very small compared to our voxel size, and therefore the effect of convolution by PSF can be ignored. In the work, we used median filter to smooth the observed image and assume the noise in the smoothed image can be considered as additive zero mean Gaussian distribution. Thus the final image formation can be represented as the following equation:

(2) $$\begin{array}{c} {\tilde{I}}_{0}\approx I+n, \end{array}$$

where ${\tilde{I}}_{0}$ and **n** represent smoothed image and image noise, respectively.

### 2.3. Image Segmentation

The purpose of image segmentation is to find a partition *ψ*(*Ω*) of the image domain *Ω* and recover the ideal image *I* as well. Under the assumption that the intensity distribution of the ideal image is piecewise constant, Chan-Vese (CV) model with level set implementation was proposed and has been widely used for image segmentation [19]. Later on, this model was further extended to global CV (G-CV) model by transforming it into a global convex optimization problem [20]. However, confocal microscopy images are characterized by intensity fall-off in depth, which makes G-CV model unsuitable for this purpose. To solve this problem, we further assume the ideal image can be described as the multiplication of an intensity piecewise constant image *c* and a depth-related bias field *b*

(3) $$\begin{array}{c} I=\sum_{i=1}^{N}\left(c_{i}\cdot u_{i}\right)\cdot b_{i}, \end{array}$$

where *N* is number of regions in the image and *u*_*i*_ is an image partition function. Under this assumption, we developed a new convex active contour model for automatic segmentation of confocal microscopy images. The model can be represented as the following equation:

(4) min⁡u∈[0,1]E(c1,c2,u)=∫Ω|∇u(x)|dx+λ∫Ω(I~0(x)−I1(x))2u(x)dx+λ∫Ω(I~0(x)−I2(x))2(1−u(x))dx.

The first term on the right side of (4) is a L1 total variation (TV) norm which is used for smoothing the variable *u*. The second and third terms are data fidelity terms which keep the intensity distribution of the ideal images close to the original image. Here, *u* is a partition variable and *λ* is a weighting constant to keep the balance among the three terms on the right side of (4). *I*_1_(*x*) = *c*_1_ · *γ* ^*z*(*x*)^ and *I*_2_(*x*) = *c*_2_ · *γ* ^*z*(*x*)^ are two ideal images that represent the intensity on two different subregions. *γ* ^*z*(*x*)^ is a depth-dependent bias field to characterize the intensity fall-off in depth property of the images, where *z* is the position of the point *x* in *z*-direction and *γ* is an experimentally determined decreasing constant.

There are total three unknown variables in our model: *c*_1_, *c*_2_, and *u*. By using first variation with respect to *c*_1_ and *c*_2_, we can obtain

(5) $$\begin{array}{c} c_{1}=\frac{\int_{x\in \Omega }u\left(x\right){\tilde{I}}_{0}\left(x\right)\gamma^{z(x)}dx}{\int_{x\in \Omega }u\left(x\right)\gamma^{2z(x)}dx},\\ c_{2}=\frac{\int_{x\in \Omega }\left(1-u\left(x\right)\right){\tilde{I}}_{0}\left(x\right)\gamma^{z(x)}dx}{\int_{x\in \Omega }\left(1-u\left(x\right)\right)\gamma^{2z(x)}dx}. \end{array}$$

To minimize (4) with respect to *u*, we adopt the fast and efficient Split Bregman method proposed in [11, 12]. Split Bregman method does not require regularization, continuation, or the enforcement of inequality constraints, and it is very efficient for solving L1-regularized optimization problems like (4). For easier description of Split Bregman method, we rewrite the form of (4):

(6) $$\begin{array}{c} \underset{u\in \left[0,1\right]}{\min}E=\int_{\Omega }\left|\nabla u\left(x\right)\right|+\lambda e_{r}\left(x\right)u\left(x\right)dx, \end{array}$$

where $e_{r}{\left(x)=({\tilde{I}}_{0}(x)-I_{1}(x)\right)}^{2}-{\left({\tilde{I}}_{0}(x)-I_{2}(x)\right)}^{2}$. Here, the term $\lambda \int_{\Omega }{\left({\tilde{I}}_{0}(x)-I_{2}(x)\right)}^{2}dx$ has been ignored because it does not include the variable *u*.

To minimize (6) with respect to *u*, we introduce an auxiliary variable *d*, such that *d* = ∇*u*. Thus, the problem of minimizing the energy function of (6) becomes to minimize the following energy function:

(7) $$\begin{array}{c} \underset{u\in \left[0,1\right],d}{\min}\int_{\Omega }\left|d\right|+\lambda e_{r}\left(x\right)u\left(x\right)dx,\;\text{with}\;\;d=\nabla u. \end{array}$$

To solve the constrained problem in (7), we use Split Bregman method. The problem becomes to solve the following sequence of optimization problems:

(8) (uk+1,dk+1)=argmin⁡u∈[0,1],d∫Ω|d|+λer(x)u(x)+μ2|d−∇u−bk|2dx,

(9) $$\begin{array}{c} b^{k+1}=b^{k}+\nabla u^{k+1}-d^{k+1}. \end{array}$$

Here, *k* = 0,1, 2,…, the third term on the right side of (8) is used to enforcing the constraint *d* = ∇*u*. *b* ^*k*^ is the Bregman vector. *μ* and *λ* are two constant weighting parameters to keep a balance of two terms. To solve (8), we adopt the alternating minimization scheme. First, we consider the minimization of (8) with respect to *u*. The minimizing solution *u* ^*k*+1^ is characterized by the optimality condition

(10) $$\begin{array}{c} \lambda \nabla u=\lambda e_{r}+\mu \mathrm{div}\left(b^{k}-d^{k}\right),\;u\in \left[0,1\right]. \end{array}$$

By using Gauss-Seidel iterative scheme, we can get an approximate solution for a 3D variable *u* ^*k*+1^. (*i* = 0,1, 2,…):

(11) ζl,m,n=dl−1,m,nx,k−dl,m,nx,k−bl−1,m,nx,k+bl,m,nx,k+dl−1,m,ny,k−dl,m,ny,k−bl−1,m,ny,k+bl,m,ny,k+dl−1,m,nz,k−dl,m,nz,k−bl−1,m,nz,k+bl,m,nz,k,ϕl,m,n=16(ul−1,m,nk+1,i+ul+1,m,nk+1,i+ul,m−1,nk+1,i+ul,m+1,nk+1,i+ul,m,n−1k+1,i+ul,m,n+1k+1,i+ζl,m,n−λμer(l,m,n)),ul,m,nk+1,i+1=max⁡{min⁡{ϕl,m,n,1},0},

where *i* is the iteration index for Gauss-Seidel iterative method and *l*, *m*, *n* are the indices of the voxel in axes *x*, *y*, and *z*, respectively. One has *u*_*l*,*m*,*n*_^*k*+1,0^ = *u*_*l*,*m*,*n*_^*k*^.

After calculating an approximate *u* ^*k*+1^, we can obtain *d* ^*k*+1^ by minimizing (8) with respect to *d*

(12) $$\begin{array}{c} d^{k+1}=\frac{\nabla u^{k+1}+b^{k}}{\left|\nabla u^{k+1}+b^{k}\right|}\max\left(\left|\nabla u^{k+1}+b^{k}\right|-\frac{1}{\lambda },0\right). \end{array}$$

Once *u* ^*k*+1^ and *d* ^*k*+1^ are available, the Bregman vector *b* ^*k*^ can be updated according to (9). For more details of 2D Split Bregman method, we refer the readers to [11].

As a summary, the procedures of using Split Bregman method to solve (7) contain the following steps.

1. Initialization: *b* ^0^, *d* ^0^ and *u* ^0^.
2. Fix *u*, calculate *c*_1_ and *c*_2_ according to (5), and further calculate *e*_*r*_.
3. Update *u* ^*k*+1^ by solving (11).
4. Update *d* ^*k*+1^ by solving (12).
5. Update *b* ^*k*+1^ by solving (9).
6. Convergence test: test whether a stable solution *u* has reached. If not, go to step (2).
7. The objects are detected by thresholding Σ = {*x* : *u*(*x*) > *α*}, where *α* ∈ [0,1]. In this paper, we choose *α* = 0.5.

## 3. Experimental Results

### 3.1. Data

In this study, we selected three groups of quail hearts. Each group had five embryonic quail hearts at the development stage of days 6, 7, 8, 9, and 14, respectively. All the hearts were processed and imaged according to Section 2.1. The image size varied from 768 × 768 × 112 (day 6) to 3075 × 2560 × 478 (day 14). To build a database of manual segmentation for reference, we invited two biologists to independently segment the hearts manually with the software ITK-SNAP [21]. Due to the large size of the image and the complexity of the heart geometry, it typically took a biologist more than one week to finish one heart segmentation. For this reason, we currently only selected one group for manual segmentation.

### 3.2. Evaluation of Automatic Segmentation

**Figure 2:**
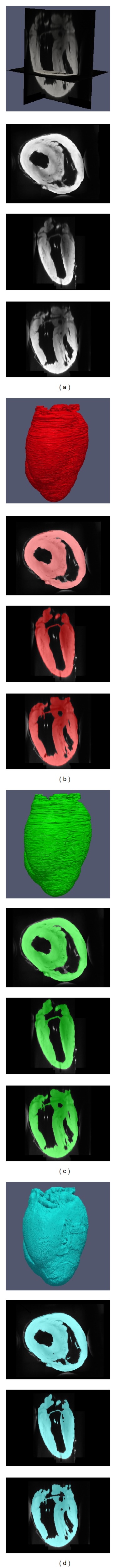
Visual comparison between manual segmentation and automatic segmentation. (a) Original 3D image and three slices in different views. (b) Manual segmentation done by the first biologist. (c) Manual segmentation done by the second biologist. (d) Automatic segmentation.

Figure 2 shows one 3D and three 2D slice views of the quail heart at day 14 with two manual segmentations and one automatic segmentation. Although the image exhibits severe intensity inhomogeneity, visual inspection of the results shows that automatic segmentation can correctly capture most of the structures of the heart as manual segmentation. The most difference between manual and automatic segmentation occurs at the regions above the atrioventricular valve which can be observed in both 3D and 2D views. Because the contrast is very low at this region, automatic segmentation algorithm only uses image information can not detect the boundary precisely, while biologists using their knowledge can manually locate the boundary. What is more, we have also observed the presence of small objects within the heart chambers only detected by automatic segmentation. These small objects could be papillary muscle that can be considered as either a part of the myocardium or a part of the blood pool. As a result, both manual and automatic segmentation for these small objects are acceptable.

**Figure 3:**
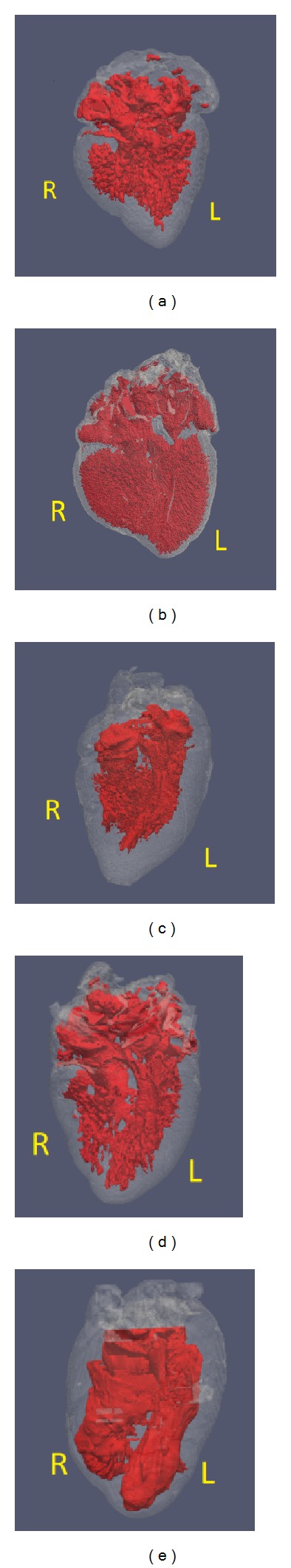
3D segmentation of one group of the hearts. Columns from left to right are the heart at days 6, 7, 8, 9, and 14. For visualization purpose, the outer boundary is rendered as transparent (L: left ventricle. R: right ventricle).

We also quantitatively evaluate our algorithm by measuring the overlap of automatic segmentation and manual segmentation by using Dice's similarity coefficient (DSC). For two segmentations *S*_1_ and *S*_2_, the DSC value is defined as 2 | *S*_1_∩*S*_2_ | /(|*S*_1_ | +|*S*_2_|). The DSC value is normalized, where 0 indicates complete dissimilarity and 1 indicates complete agreement. The overlap values reflecting the variability between the manual segmentations by two biologists are listed in the second row of Table 1. Except for days 6 and 7, all the DSC values are greater than 0.85, which means there is sufficient level of reliability for the two manual segmentations. The reason of low DSC values at days 6 and 7 is that the geometry of the hearts at these days is very complex as shown in Figure 3, and thus it is difficult for the biologists to achieve high agreement. The overlap comparison between automatic and manual segmentations is listed in the third and fourth rows of Table 1. Similarly, we can find the DSC values are low at days 6 and 7 and high at the rest. Overall, the overlap measures show that the automatic segmentation method has similar level of variability to the manual segmentation, which means our automatic segmentation method is applicable to EHM study.

### 3.3. EHM Study

Figure 3 shows automatic segmentation results of one group of hearts. As we can see, an obvious phenomenon of early heart development is the morphology evolution of the ventricles. The left and right ventricles are merged together and exhibit sponge network structure at days 6 and 7 as the result of cardiac looping [4]. The interventricular septum starts to grow between day 7 and day 8, as the ventricles are partially separated at day 8. At day 9, the interventricular septum eventually forms and divides the ventricles into the left and right ventricle. However, the two ventricles still present some sponge structure at day 9. The shape of the ventricles eventually becomes mature at day 14.

We also quantify the average volume of the whole heart and the luminal space at different stages of development based on the segmentation results. We use the open source VTK library (http://www.vtk.org/) to calculate the average volume and list the average volume values in Table 2. We find that the average volume of the whole heart and the luminal space increased from 2.6 mm^3^ to 77.5 mm^3^ and 0.41 mm^3^ to 20.2 mm^3^, respectively, which is nearly two orders of magnitude increase in an incubation period of approximately 10 days. This finding is similar in range to the findings in [8]. Furthermore, we also find that the average volume of the whole heart grows faster than the luminal space, which means that the myocardium grows towards both inside and outside.

## 4. Discussion

The technique outlined in this paper provided the framework of imaging and automatic segmentation of developing hearts for EHM study. By combining confocal microscopy imaging with optical clearing, our method was able to achieve penetration depth over 6 mm that enabled us to acquire volumetric images of the developing heart through the whole incubation period. We believe this imaging data can help biologists to understand more details of early heart development and investigate events that lead to congenital heart defects.

Image segmentation is always a headache for researchers in this field because of the complexity of the developing geometry. The convex active contour model proposed in this paper was a first step towards automatic segmentation in EHM study and showed promising results. One significant challenge in developing heart segmentation is the lack of a gold standard. Due to the expensive labor cost to label the images, we provided limited validation in the paper. In the future, we will build a larger manually segmented database for segmentation algorithm validation. What is more, this database could also be used for training and testing parameter-free machine learning algorithms.

The ultimate goal of this work will be heart growth modeling. Due to the complexity of developing heart, current heart growth modeling mainly focuses on very early stages of EHM [22]. To the best of our knowledge, there exist no works on modeling the whole EHM process from the single tube shape to four-chambered shape. With EHM knowledge from EHM study, we will work towards data-driven heart growth modeling.

## 5. Conclusion

We proposed an imaging approach and a novel automatic segmentation method for EHM study. We demonstrated the applicability of our imaging method to capture the 3D structure of embryonic quail hearts and also proved the efficiency of our segmentation algorithm for EHM study in both visual inspection and quantitative analysis. Based on the findings from EHM study, we believe this work could help us to further understand the fundamental mechanisms of embryonic heart development.

## Figures and Tables

**Table 1:** DSC values that measure the overlap between the two manual segmentations, the first manual segmentation against automatic segmentation and the second manual segmentation against automatic segmentation.

	Day 6	Day 7	Day 8	Day 9	Day 14
Biologist 1 versus biologist 2	0.75	0.79	0.85	0.87	0.93
Automatic versus biologist 1	0.65	0.72	0.78	0.85	0.88
Automatic versus biologist 2	0.68	0.75	0.79	0.81	0.91

**Table 2:** Average volume of the whole heart and the luminal space at different stages of development (mm^3^).

	Day 6	Day 7	Day 8	Day 9	Day 14
Total heart	2.6	3.4	6.0	10.2	77.5
Luminal space	0.41	0.62	0.75	1.74	20.2

## References

[1] Icardo J, Manasek F (1992). Cardiogenesis: development mechanisms and embryology. *The Heart and Cardiovascular System*.

[2] World Health Organization (2011). *Global Atlas on Cardiovascular Disease Prevention and Control*.

[3] Lacktis J, Manasek F (1978). An analysis of deformation during a normal morphogenic event. *Morphogenesis and Malformation of the Cardiovascular System*.

[4] Taber LA, Keller BB, Clark EB (1992). Cardiac mechanics in the stage-16 chick embryo. *Journal of Biomechanical Engineering*.

[5] Liebling M, Vermot J, Fraser SE Double time-scale image reconstruction of the beating and developing embryonic zebrafish heart.

[6] Groenendijk BCW, Hierck BP, Vrolijk J (2005). Changes in shear stress-related gene expression after experimentally altered venous return in the chicken embryo. *Circulation Research*.

[7] Liebling M, Forouhar AS, Wolleschensky R (2006). Rapid three-dimensional imaging and analysis of the beating embryonic heart reveals functional changes during development. *Developmental Dynamics*.

[8] Butcher JT, Sedmera D, Guldberg RE, Markwald RR (2007). Quantitative volumetric analysis of cardiac morphogenesis assessed through micro-computed tomography. *Developmental Dynamics*.

[9] Jenkins MW, Rothenberg F, Roy D (2006). 4D embryonic cardiography using gated optical coherence tomography. *Optics Express*.

[10] Smith B (2001). Magnetic resonance microscopy in cardiac development. *Microscopy Research and Technique*.

[11] Goldstein T, Osher S (2009). The split Bregman method for L1 regularized problems. *SIAM Journal on Imaging Sciences*.

[12] Mao H, Liu H, Shi P A convex neighbor-constrained active contour model for image segmentation.

[13] Fishbaugh J, Prastawa M, Durrleman S, Piven J, Gerig G (2012). Analysis of longitudinal shape variability via subject specific growth modeling. *Medical Image Computing and Computer Assisted Intervention*.

[14] Durrleman S, Pennec X, Trouve A, Gerig G, Ayache N (2009). Spatiotemporal atlas estimation for developmental delay detection in longitudinal datasets. *Medical Image Computing and Computer Assisted Intervention*.

[15] Fong KW, Toi A, Salem S (2004). Detection of fetal structural abnormalities with US during early pregnancy. *Radiographics*.

[16] Genina EA, Bashkatov AN, Tuchin VV (2010). Tissue optical immersion clearing. *Expert Review of Medical Devices*.

[17] Smith RM, Matiukas A, Zemlin CW, Pertsov AM (2008). Nondestructive optical determination of fiber organization in intact myocardial wall. *Microscopy Research and Technique*.

[18] Dey N, Blanc-Feraud L, Zimmer C (2006). Richardson-Lucy algorithm with total variation regularization for 3D confocal microscope deconvolution. *Microscopy Research and Technique*.

[19] Chan TF, Vese LA (2001). Active contours without edges. *IEEE Transactions on Image Processing*.

[20] Bresson X, Esedoglu S, Vandergheynst P, Thiran J-P, Osher S (2007). Fast global minimization of the active contour/snake model. *Journal of Mathematical Imaging and Vision*.

[21] Yushkevich PA, Piven J, Hazlett HC (2006). User-guided 3D active contour segmentation of anatomical structures: significantly improved efficiency and reliability. *NeuroImage*.

[22] Goenezen S, Rennie M, Rugonyi S (2012). Biomechanics of early cardiac development. *Biomechanics and Modeling in Mechanobiology*.

